# Neurocognitive functions and brain atrophy after proven neuroborreliosis: a case-control study

**DOI:** 10.1186/s12883-015-0386-1

**Published:** 2015-08-19

**Authors:** Holger Schmidt, Marija Djukic, Klaus Jung, Manfred Holzgraefe, Peter Dechent, Nicole von Steinbüchel, Joachim Blocher, Helmut Eiffert, Carsten Schmidt-Samoa

**Affiliations:** University Medical Centre Göttingen, Department of Neurology, Robert-Koch-Str. 40, Göttingen, 37075 Germany; University Medical Centre Göttingen, Department of Cognitive Neurology, Robert-Koch-Str. 40, Göttingen, 37075 Germany; Ev. Hospital Göttingen-Weende, Department of Geriatrics, An der Lutter 24, Göttingen, 37075 Germany; University Medical Centre Göttingen, Department of Medical Statistics, Humboldtallee 32, Göttingen, 37073 Germany; Asklepios Schildautal-Hospital Seesen, Department of Neurological Rehabilitation, Karl-Herold-Str. 1, Seesen/Harz, 38723 Germany; University Medical Centre Göttingen, Department of Medical Psychology and Medical Sociology, Waldweg 37, Göttingen, 37073 Germany; University Medical Centre Göttingen, Department of Medical Microbiology, Kreuzbergring 57, Göttingen, 37075 Germany; University Medical Centre Göttingen, Department of Neuropathology, Robert-Koch-Str. 40, Göttingen, 37075 Germany; Elbe Kliniken Stade-Buxtehude, Department of Neurology, Bremervörder Str. 111, Stade, 21682 Germany

**Keywords:** Lyme, Neuroborreliosis, Neuropsychological deficits, Neurological deficits

## Abstract

**Background:**

Patients often report neurocognitive difficulties after neuroborreliosis (NB). The frequency and extent of cognitive problems in European patients have been studied incompletely.

**Methods:**

Sixty patients received a neurological and neuropsychological work-up 6 months or longer after treatment for proven NB. Quality of life, psychiatric symptom load, and brain atrophy were measured. All results were compared with a group of 30 healthy control persons adapted for age, gender and education being serologically negative for *Borrelia burgdorferi senso latu*. A cognitive sum score and a global sum score including cognitive, psychological results and quality of life data was calculated for both groups.

**Results:**

Patients after NB showed a lower (i.e. more impaired) score on the Scripps Neurological rating scale (SNRS), but the observed neurological deficits were generally mild (mean ± SD: 97.1 ± 4.7 vs. 99.1 ± 2.4, p = 0.02). The mean neuropsychological domain results of the NB group were all within the normal range. However, a lower performance was found for the frontal executive function z-values (mean ± SD –0.29 ± 0.60 vs. 0.09 ± 0.60; p = 0.0059) of NB patients. Comparing the global sum score (mean ± SD 11.3 ± 4.2 _*NB*_ vs. 14.3 ± 2.9 _*control*_, p = 0.001) and the cognitive sum score of the NB group with those of the control group (mean ± SD -0.15 ± 0.42 _*NB*_ vs. 0.08 ± 0.31 _*control*_, p = 0.0079), both differences were statistically different. The frequencies of impaired global sum scores and those of the pathological cognitive sum scores (p = 0.07) did not differ statistically. No significant differences were found for health-related quality of life (hrQoL), sleep, psychiatric symptom load, or brain atrophy.

**Conclusion:**

The mean cognitive functions of patients after proven NB were in the normal range. However, we were able to demonstrate a lower performance for the domain of frontal executive functions, for the mean cognitive sum score and the global sum score as a sign of subtle but measurable sequelae of neuroborreliosis. Brain atrophy is not a common consequence of neuroborreliosis.

## Background

There are only few studies on cognitive symptoms in patients who have had the European form of neuroborreliosis (NB). This gap in our knowledge gives rise to speculations about the clinical course of NB, often leading to the impression that NB inevitably results in severe neurocognitive sequelae. Population-based studies clarified the low probability of developing either a chronic form of NB [1]. In a study with 20 patients with previously diagnosed NB, Benke et al. [2] found significantly poorer verbal learning and memory functions paired with problems concerning the executive functions as residual symptoms after NB. The second, more recent study from Scandinavia applying a short test battery of four neuropsychological tests demonstrated significantly lower mean results in the NB group while the extent of the impairment was rather mild [3]. In a similar study from the U.S. with 44 patients, however, 36 % showed impaired scores when tested with a naming test compared to 14 % of healthy control persons [4]. Whether the difference between the studies is due to the divergent forms of neuroborreliosis in Europe and America, or whether it only represents differences in methodology remains to be clarified. In addition, our study aims to elucidate the extent and frequency of brain atrophy caused by NB as reported by Tarasow et al. in 60 % of their patients [5].

## Methods

### Patients

**Fig. 1:**
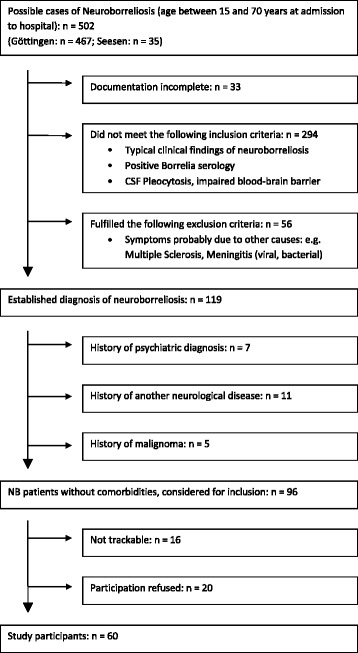
Study exclusion tree

We reviewed the clinical files of patients from two neurological centres (University Medical Centre Göttingen and Asklepios Klinik Schildautal/Seesen, both Germany) who had been diagnosed and treated for NB from 1994 to 2009. Patients had to be between 15 and 70 years of age at the time of treatment. Patients with other neurological or psychiatric diseases (including alcohol or any substance abuse) were excluded. Eligible NB patients had to show a documented typical clinical presentation of NB in combination with positive serum antibodies against Borrelia burgdorferi sensu lato and either a cerebrospinal fluid (CSF) pleocytosis with an elevated Borrelia-specific CSF-to-serum antibody index (AI) or a CSF pleocytosis accompanied by an erythema migrans (EM) at the time of presentation. All eligible former patients were invited by mail. The treatment had to be completed for at least six month to participate in this study. Sixty patients participated. According to our power calculations which were based on a paper published by McAuliffe and colleagues [6], a sample size of 10 per group would have been sufficient to reach a statistical power of 80 % for the cognitive sum score. With the sample size of 60, the statistical power of the presented calculations is estimated > 90 %. All patients had completed their antibiotic treatment regimen according to the German guidelines for neuroborreliosis. Most of them were treated with ceftriaxone (*n*=51), seven with penicillin G, and two with doxycycline. The exclusion tree is given in Fig. 1. The control group consisted of 30 neurologically healthy, serologically Borrelia-negative volunteers. This group was adapted for gender (f/m = 20/40 _*NB*_ vs. 12/18 _*Control*_, *p*=0.64), age (mean ± SD 46.9 ± 13.9 _*NB*_ vs. 42.3 ± 16.0 _*Control*_ years, *p*=0.19), and years of education (10.6 ± 1.7 _*NB*_ vs. 11.2 ± 1.8 _*Control*_ years, *p*=0.14). Likewise, marital status and occupational situation in the control group and the patient group did not differ (Table 1). The control population was recruited by putting bulletins at several public pin boards. As a compensation for their efforts, all participants (also those from the NB group) received a refund of travel expenses.

**Table 1:** Comparison of baseline parameters between the two study groups

Parameter	Control group (*n*=30)	NB group (*n*=60)	p (univariate)	p (multivariate)
Age [years]	42.3 ± 16.0	46.9 ± 13.9	0.1862	-
	42.5 (19.0–72.0)	46.0 (18.0–71.0)		
Gender			0.6413	-
Female	11 (40 %)	20 (33.3 %)		
Male	18 (60 %)	40 (66.7 %)		
Family status			0.5106	-
unmarried	4 (13.3 %)	10 (16.7 %)		
married	16 (53.4 %)	39 (65.0 %)		
allied	8 (26.7 %)	9 (15.0 %)		
divorced	1 (3.3 %)	1 (1.7 %)		
widowed	1 (3.3 %)	1 (1.7 %)		
Education [years]	11.2 ± 1.8	10.6 ± 1.7	0.1428	-
	12.0 (8.0–13.0)	10.0 (8.0–13.0)		
Full-time work			0.8233	-
no	16 (53.3 %)	29 (48.3 %)		
yes	14 (46.7 %)	31 (51.7 %)		
Part-time work			1.0	
no	25 (83.3 %)	50 (83.3 %)		
yes	5 (16.7 %)	10 (16.7 %)		
Unemployed			0.5506	-
no	30 (100.0 %)	58 (96.7 %)		
yes	2 (3.3 %)	2 (3.3 %)		
Retired			1.0	-
no	26 (86.7 %)	53 (88.3 %)		
yes	4 (13.3 %)	7 (11.7 %)		

Descriptive values are means ± standard deviations and medians (minima-maxima)

The study was approved by the Ethics Committee of the University Medical Centre Göttingen, and all participants gave their written informed consent.

### Examination

Both groups received a thorough standardized neurological examination quantified with the Scripps neurological rating scale (SNRS) [7], a neuropsychological evaluation including measurements for attention (Testbatterie zur Aufmerksamkeitsprüfung, TAP) [8] (subtests selective attention (alertness); stimulus selectivity (go-no-go) and divided attention)). Short-term and working memory were assessed with the German version of the Wechsler Memory Scale-R (WMS-R) (subtests digit span forward/backward (fw/bw); block span fw/bw; logical memory (LM), part I) [9], the first trial of the California Verbal Learning Test (CVLT) [10] and the two-back task from the TAP battery [8]. Verbal learning and long-term memory were tested with WMS-R (subtests verbal paired associates; LM, part II) [9], CVLT [10] and with the Verbal Learning Test (VLT) [11].

Non-verbal learning and memory were measured with the WMS-R (subtest visual paired associates) [12], the Rey-Osterrieth complex figures task (ROCF) [13], the Non-Verbal Learning Test (NVLT) [14], Lern- und Gedächtnistest 3 (LGT-3) (subtest city map) [15]. Executive functions/psychomotor speed were examined by verbal fluency tasks Regensburger Wortflässigkeitstest (RWT) [16] (subtests lexical fluency with and without alterations, subtests semantic fluency with and without alterations), figural fluency task (FF) [17], a verbal concept formation task from Hamburg-Wechsler Intelligenztest für Erwachsene-R (HAWIE-R); subtest similarities (Gemeinsamkeiten finden) [12], CVLT clustering and a figural concept formation task, Wisconsin Card Sorting Test (WCST) [18], and the tower of Hanoi test [19]. Visuo-constructive functions were examined with the HAWIE-R (subtest Mosaic test [12]) and the ROCF score for figure copying [13]. Z-values were calculated for both groups according to the respective test manuals before group comparisons. Domain scores for each subject were calculated based on the mean test results of all tests from the respective domain. Tests were defined as pathologically impaired if the z-value was < -1.5. A domain was judged as pathological if more than half of the included tests of the respective domain showed z-values below -1.5.

### Psychiatric symptoms

Scores for depressive mood, anxiety, fatigue, fibromyalgia symptoms and the global psychiatric symptom load were measured with the German versions of the following tools: Beck’s Depression Inventory (BDI) [20], Hamilton Depression Scale (HAMD) [21], and Symptom Checklist 90R (SCL-90R) [22], the Hospital Anxiety and Depression Scale (HADS-D) [23], State-Trait-Anxiety Inventory (STAI X-1 and X-2) [24], the Fibromyalgia Impact Questionnaire (FIQ) [25], and the Functional Assessment of Chronic Illness Therapy-Fatigue (FACIT-Fatigue) [26].

### Health-related quality of life (hrQoL)

Self-assessment questionnaires focused on the participant’s global satisfaction (Satisfaction of Life Scale [27], SF-36 [28]), and sleep quality (Pittsburgh Sleep Quality index (PSQI) [29].

All self-assessment questionnaires were sent to the participant after the first telephone contact, i.e. before clinical presentation, to allow him/her to fill in the answers in a familiar environment.

### Brain atrophy

Fifty-four out of sixty former patients and all of the control persons agreed to be examined by MR tomography. A standardized protocol for magnetic resonance (MR) imaging of the brain was used to determine the individual brain atrophy in relation to the skull volume. The normalized volumes of the whole brain, grey matter, white matter, peripheral grey matter, and the inner CSF volume were measured with the voxel-based method FSL-Sienax. High resolution T1-weighted MR scans of the brain (3D turbo fast low angle shot (FLASH), 1 mm ^3^ isotropic resolution, repetition time: 2250 ms, inversion time: 900 ms, echo time: 3.26 ms, flip angle: 9 deg, 3 Tesla (Magnetom TIM Tio, Siemens Healthcare, Erlangen, Germany) served as the data source. In short, the Sienax technique references the above-mentioned segmented tissue volumes to the size of the skull. Size-corrected segmented volumes are in turn compared to the respective volumes derived from a normalized standard brain. Thus an estimation of brain atrophy can be obtained with one punctual measurement [30].

### Statistical methods

Categorial parameters were compared between the two groups by Fisher’s exact test and metric parameters (e.g. domain means) by either the t-test or the Mann-Whitney U-Test. The t-test was employed when data were normally distributed, which was checked by quantile-quantile plots. In order to further evaluate the clinical relevance of these differences, we compared patients and healthy controls with respect to the existence of pathological cognitive domains (i.e., domains with more than half of the tests showing pathological results, defined as z-values < -1.5).

In order to enable a comparison with other European results, we also used an algorithm proposed by Eikeland et al. [3] to build a sum score incorporating 19 of the subtasks (Table 2): The individual sum scores were further categorized with respect to the mean sum score of the control group. In detail, three categories were formed: normal (≤ 1 SD below the mean sum score of the control group), deficit (> 1, ≤ 2 SD below the mean sum score of the control group) and impaired (> 2 SD below the mean sum score of the control group). The distribution of the three categories was compared between NB and control group by Fisher’s exact test. Those parameters which were significantly different in the univariate analysis were combined in a multivariate logistic regression analysis to assess the independence of the difference. Frequencies were compared with a two-tailed Fisher’s exact test. All tests were carried out using the free software R (version 2.8, http://www.r-project.org) with a significance level of *α*< 5 %.

**Table 2:** Comparison of cognitive and neuropsychiatric data between the two study groups

Parameter	Control group (*n*=30)	NB group (*n*=60)	p (univariate)	p (multivariate)
State-trait Anxiety Inventory	31.7±4.5	33.7±6.3	0.0761	-
(STAI) State (X1)	31.0 (22.0–44.0)	33.4 (21.0–53.0)		
**State-trait Anxiety Inventory (STAI) Trait (X2)**	**31.2** ±**6.8**	**35.3** ±**10.6**	**0.0345**	**n.s.**
	**30.0 (21.0–49.9)**	**32.0 (20.0–67.0)**		
**Hamilton Depression Scale**	**4.6** ±**5.9**	**8.6** ±**8.0**	**0.010**	**n.s.**
	**3.0 (0.0–20.0)**	**6.0 (0.0–28.0)**		
**HADS A**	**3.1** ±**2.8**	**3.1** ±**2.8**	**0.0190**	**n.s.**
	**3.0 (0.0–11.0)**	**4.0 (0.0–15.0)**		
HADS D	2.4±3.3	3.9±3.8	0.0737	-
	1.0 (0.0–12.0)	3.0 (0.0–15.0)		
Sleep quality (PSQI)	4.8±3.6	4.5±3.0	0.7454	-
	4.5 (0.0–14.0)	4.0 (0.0–15.0)		
Diener’s Quality of Life Score	20.4±3.5	19.2±4.3	0.1919	-
	20.0 (9.0–25.0)	20.0 (8.0–25.0)		
Quality of Life Bar	7.4±2.2	6.4±2.2	0.0615	-
	8.1 (1.9–10.0)	7.0 (1.1–9.7)		
**Nottingham Activities of Daily Living (NADL)**	**87.8** ±**1.3**	**86.8** ±**2.1**	0.0135	**n.s.**
	**88.0 (81.0–88.0)**	**88.0 (81.0–88.0)**		
Beck’s Depression Index (BDI)	3.1±3.6	4.9±6.5	0.1087	-
	2.0 (0.0–15.0)	2.0 (0.0–15.0)		
**Fibromyalgia Impact Questionnaire (FIQ)**	**6.2** ±**7.2**	**17.9** ±**19.8**	**0.0001**	**n.s.**
	**3.2 (0.0–35.0)**	**10.4 (0.0–78.6)**		
Working memory	−0.09±0.55	−0.27±0.56	0.1525	-
	−0.01 (−1.54–0.85)	−0.26 (−1.38–1.08)		
Verbal memory	−0.11±0.58	−0.37±0.67	0.0603	-
	−0.07 (−1.47–0.83)	−0.27 (−1.83–0.88)		
Non-verbal memory	0.08±0.61	−0.13±0.50	0.1037	-
	0.23 (−1.61–1.34)	−0.10 (−1.36–0.84)		
Visuo-constructive functions	0.62±0.59	0.42±0.78	0.1771	-
	0.62 (−0.44 –1.72)	0.57 (−1.77–1.81)		
**Frontal executive functions**	**0.09** ±**0.60**	**−0.29** ±**0.60**	**0.0059**	**0.0161**
	**0.21 (−1.43–1.19)**	**−0.22 (−1.62–1.04)**		
Alertness	−0.17±0.39	−0.23±0.45	0.5427	-
	−0.16 (−1.16–0.68)	−0.21 (−1.23–0.75)		-
**Cognitive sum score**	**0.08** ±**0.31**	**−0.15** ±**0.42**	**0.0079**	**0.01**
	**0.1 (−0.66–0.78)**	**−0.12 (−1.03–0.93)**		
SCL 90r GSI	41.3±11.8	46.6±14.9	0.0772	-
	42.0 (21.0–74.0)	45.0 (23.0–75.0)		
SCL 90r PST	40.9±11.3	45.8±14.3	0.0852	-
	41.0 (21.0–74.0)	44.0 (23.0–79.0)		
**Global sum score**	**0.7** ±**0.30**	**−0.15** ±**0.42**	**0.0083**	-
	**0.10 (−0.66–0.78)**	**−0.12 (−1.03–0.93)**		

Descriptive values: means ± standard deviations and medians (minima-maxima)

Bold face: Significant differences

## Results and discussion

### Results

#### Neurological examination

The diagnosis and treatment of the former NB patients dated back 4.5 ± 3.7 years (mean ± SD). When they presented with neuroborreliosis, patients were 42.9 ± 14.4 years old; 39 % complained of headaches, 10 % experienced fever, in 20 % signs of meningitis were present (mostly end-grade nuchal rigidity), 44 % showed a facial palsy, in 66 % radicular symptoms were predominant, 7 % reported cognitive alterations, and in 7 % an altered mood was observed. Only 45 % remembered a tick bite before the first symptoms of NB began. Forty percent had noticed local erythema. 12.5 % developed the neurological symptoms that led to hospital admission within several days. The majority of NB patients experienced a subacute course, 53.6 % developing the symptoms gradually within 14 days. In 28.6 % symptom development took up to two month until diagnosis, and a minority of patients (5.3 %) had neurological symptoms for more than two months before the diagnosis was made. One patient with radicular pain, who waited more than one year before the correct diagnosis was made, suffered at the time of presentation from a chronic myelitic NB and kept residual symptoms, especially urine incontinence. On re-examination, 56 % of the NB group displayed (mostly subtle) neurological signs resulting in a significantly lower SNRS when compared to the healthy control group (97.1 ± 4.8 vs. 99.3 ± 2.2, *p*=0.02). Of the patients with residual neurological signs, 79 % (44 % of all patients) were in the range of “mild impairment” (SNRS score range 93 to 99), 15 % (8 %) could be grouped into “moderate impairment” (SNRS score range 92 to 86), and only two patients (6 % of those with neurological symptoms; 3 % of all patients) remained severely impaired (SNRS ≤ 85) according to the SNRS grading used by the IFNB MS study group [31]. In all patients, the symptoms could at least be explained as residua of the initial symptoms of NB. However, due to the retrospective design of the study with it’s long interval between disease and follow-up examination, other contributing factors eventually forgotten or not mentioned by the participants cannot be excluded.

The most frequent abnormalities found in the neurological examination were sensory symptoms, especially radicular pain or hypaesthesia and residual facial asymmetry. In detail, of the 33 patients with neurological symptoms at reexamination, 45 % (25 % NB; 7 % Control) had mild facial palsy, 48 % (27 % NB; 11 % Control) showed sensory deficits, judged to be mild in all but one patient who was rated as being moderately impaired. Motor impairment was found in 21 % of patients with neurological symptoms (12 % NB, 0 % Control), again judged to be mild in all but one patient. Mild reflex differences were present in 30 % of the patients with residual neurological signs of NB (17 % NB; 11 % Control). A positive Babinski sign was found in one NB patient. Mild cerebellar signs were recorded for 12 % in the patients with neurological symptoms (7 % NB; 0 % Control). Gait and balance were found to be impaired in 27 % of NB patients with neurological symptoms (15 % NB, 4 % Control); the impairment was judged to be mild in 7, moderate in 2 patients. 3 patients reported problems with bladder/bowel functioning (5 % NB; 0 % Control). Mood alterations were recorded in 4 patients (7 % NB; 0 % Control). Cranial nerve signs were present in 12 % of patients with neurological impairment (7 % NB; 4 % Control).

The scores of the SNRS at re-examination were negatively correlated with the patients’ age at admission and with the interval from the onset of symptoms of NB until the start of antibiotic therapy, i.e. the older patients and the longer the delay in treatment, the poorer the neurological outcome score (Spearman’s *r* = –0.34, *p* = 0.01, and *r* = –0.32, *p* = 0.02).

#### Neuropsychological evaluation

**Fig. 2:**
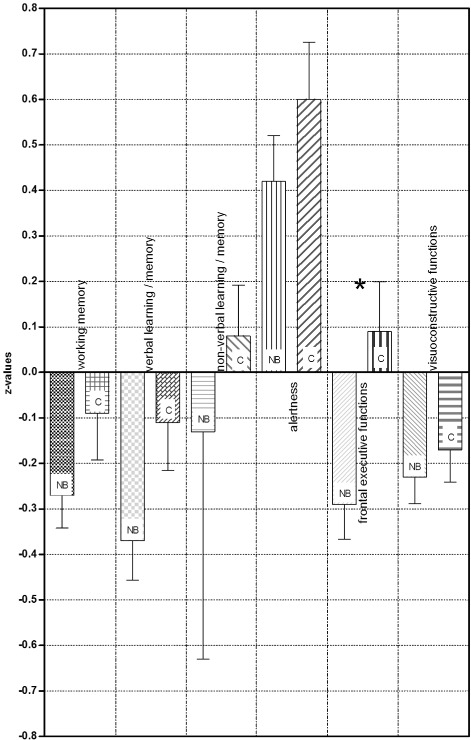
Cognitive domain results. NB: Neuroborreliosis groups; C: control group; asterisk: significant differences

The detailed results test by test are given in Table 2. In all domains the patient group’s mean z-values were lower than in the control group (Fig. 2). However, all group means fell within the normal range, and a statistically significant difference was only present in the domain of frontal executive functions (–0.29 ± 0.60 _*NB*_ vs. 0.09 ± 0.60 _*Control*_, *p*=0.006). The independence of this observation could be confirmed by logistic regression analysis (*p*=0.016). In addition, the difference between both cognitive sum scores showed a statistical difference (11.3 ± 4.2 _*NB*_ vs. 14.3 ± 2.9 _*Control*_, *p*=0.001). Based on a cut-off value of z < -1.5 for the domains’ means, we did not find any resulting pathological mean (working memory, non-verbal learning/memory, or alertness) for either group (Table 3).

**Table 3:** Frequency of pathological performances per domain and group

Parameter	Control	NB	p (Fisher two-tailed
	group ^⊕^	group	exact test)
Working memory	0/30	0/60	-
Verbal memory	0/30	6/60	0.173
Non-verbal memory	0/30	1/60	1.0
Visuo-constructive functions	0/30	0/60	-
Frontal executive functions	2/30	1/60	0.257
Cognitive sum score	0/30	0/60	1.0

^⊕^Subjects with more than 50 % pathological (z ≤ -1.5) scores per domain

**Fig. 3:**
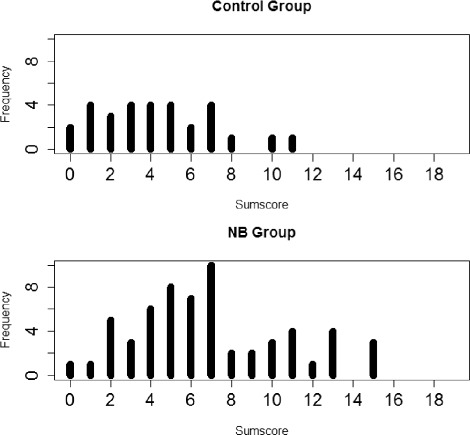
Distribution of congnitive sum scores. NB: Neuroborreliosis groups; C: control group

For the frequencies of pathological domains, none of the differences between patient and control group differed significantly (verbal learning and memory: *n* = 6/60 _*NB*_ vs. *n* = 0/30 _*Control*_, *p*=0.173; visuo-constructive functions *n* = 5/60 _*NB*_ vs. *n* = 0/30 _*Control*_, *p*=0.165; frontal executive functions *n* = 1/60 _*NB*_ vs. 0/30 _*Control*_, *p*=1.00). Using the method of Eikeland et al., 15 sum scores of the NB patients but only two sum scores of persons belonging to the Control group were classified as impaired (Table 4) when comparing the frequencies of all three catergories (“normal”, “impaired”, “defect”) of the two groups with each other by means of the Chi2-test (*p*=0.07). However, comparing the frequencies of impaired test results according to Eikeland et al. in the NB group with those in the control group, Fisher’s exact test showed a significant difference, i.e. a more frequent occurence of a deficit in the NB group (*p*=0.0435) (see also Fig. 3). There were no correlations between the scores for anxiety, depressive mood, sleep quality, general quality of life or psychiatric symptom load with the z-values of the neuropsychological domains.

**Table 4:** Frequencies of categorized global sum scores per group

Sum score	NB group	Control group	p(Chi ^2^-Text
categories	(*N*=60)	(*N*=30)	
Normal	41 (68 %)	27 (90 %)	0.07
Deficit	4 (7 %)	1 (7 %)	
Impairment	15 (25 %)	2 (3 %)	

Categories: Normal: z ≥-1, Deficit: z = <-1 and = z ≥-2, Impaired < z = -2

#### Questionnaires for psychiatric symptom load, hrQoL, activities of daily living

For all questionnaires except the PSQI questionnaire, patients after NB showed z-values with a trend towards more impairment than the control group. However, most of the NB patients recovered well and the mean results did not exceed the normal range. The differences between the NB group and the control group were significant for the anxiety subscale of the Hospital Anxiety Scale-A (HADS-A), the STAI-X2 scale, and the Hamilton Depression Scale. Patients after NB also displayed significantly higher scores in the Fibromyalgia Impact Questionnaire than the healthy control group.

The NADL score was lower (i.e. “more impaired”) in patients after NB in the univariate analysis. Although we found these group differences, all mean values fell within the normal range and were far from being pathological. Using the multivariate analysis, none of the above mentioned univariate differences yielded significant differences (see Table 2). The BDI correlated significantly (*r* = 0.3; *p* = 0.03) with the time interval from onset of symptoms to treatment. In contrast, we could not detect any significant correlations between the scores for depression and the cognitive domains.

#### Measures of brain atrophy

Neither the normalized brain volumes, nor the inner CSF spaces, nor the white or grey matter volumes differed between the groups (Table 5).

**Table 5:** Comparison of the volumetric cerebral MR analysis of the two study groups

Parameter	Control group (*n*=30)	NB group (*n*=54)	p
Peripheral grey matter [ml]	621.4±50.4	618.5±47.2	0.8118
	624.4 (514.7–719.7)	619.7 (518.7–764.4)	
Ventricular volume [ml]	42.0±15.7	42.4±19.3	0.9516
	40.1 (19.9–8.5)	38.9 (17.5–13.6)	
Total grey matter [ml]	818.0±65.1	811.6±60.6	0.6642
	817.3 (672.1–943.2)	812.2 (690.5–991.1)	
Total white matter [ml]	691.0±42.6	691.5±36.7	0.9145
	687.9 (566.9–763.5)	690.5 (623.2–781.5)	
Total brain volume [ml]	1509.0±89.8	1503.1±79.3	0.8409
	1511.0 (1298.0–1681.0)	1513.0 (1324.0–1679.0)	

Descriptive values are means ± standard deviations and medians (minima-maxima)

### Discussion

The cognitive residuals of proven NB in Europe have been examined in only two studies [2, 3], reporting considerable cognitive long-term sequelae. A study from the U.S. found poorer cognitive functioning after NB as well, but the differences did not reach statistical significance [32]. In the present study we demonstrate, with a sufficient number of patients, that after proven NB the mean performance in frontal executive functions is significantly poorer than in the control group, and that impaired cognitive sum scores are observed significantly more frequently in the NB than in Control group subjects. In addition, patients after NB were significantly more anxious, complained more often of muscle pain and had lower scores (i.e. were more impaired) in their standardized neurological examination. All these differences, however, were only group differences. The mean results for the NB group were all in the normal range, far from being pathological. The comparison of the frequencies of impaired cognitive sum scores according to Eikeland et al. did not yield a statistical difference [3]. Looking at the frequencies of z-values < –1.5 per domain in individual persons, there were no statistical differences between the control and patient group. This is in line with several studies focusing on the global clinical and neurological outcome after NB [33–36].

Taken together, the patients in our study - after adequately treated NB - had a distinctively more favourable outcome than those of the older comparable study [2] and a slightly better outcome when compared to the newer study from Scandinavia [3]. In both of the mentioned studies significant and much more important problems were observed in the domains of verbal learning/memory, attention and executive functions after NB. The reason for this discrepancy might be the smaller group size that was examined in both trials resulting in a possible selection bias. Another reason for the significantly poorer outcome of the patients of Benke et al., however, might also be the interval of 15 years between the studies. Today’s awareness on the part of medical doctors and patients with regard to signs of NB might have grown, possibly leading to earlier presentation, earlier diagnosis and thus earlier and more effective treatment than in the past. This might also be reflected by the fact that most of our patients sought medical treatment within 14 days after the onset of symptoms. Although cognitive results in our patients were not negatively correlated with the delay of treatment, this might not be transferable to geographic areas where people tend to wait longer with their presentation. The more recent previous study, however, was performed with a sufficient number of patients but compared the raw scores of the tests instead of age, gender and education-corrected z-values of their data.

Our interpretation of the present data is that functionally relevant cognitive deficits after proven NB are rare exceptions and not the rule. However, also in our analysis, there were more participants from the NB group below a cognitive sum score of z < –2 than in the healthy control group (8 of 60 vs. 2 of 30). This difference did not, however, reach statistical significance in contrast to the study from Scandinavia [3].

The results of the cognitive examinations in our NB group compare well with the questionnaires that addressed psychological symptoms, quality of life, and activities of daily living; again, with the exception of sleep quality, we observed a higher symptom load in NB patients, but the mean values for the symptom load were all in the normal range. Similar results were found in the Scandinavian population examined by Eikeland and co-workers (even though they interpreted their findings differently with respect to clinical relevance) [37]. As in the study of Krupp et al. [38], who examined patients after the North American form of NB, the cognitive functions in our European study population did not correlate with the scores of the depression questionnaires.

These findings are in contrast to the often severe cognitive and psychiatric complaints of patients referred to neurological departments because of suspected NB, who display positive serologic tests but not the typical signs for NB in the CSF [39]. Despite the described difference for the frontal executive functions in NB patients compared to control persons that finally led to the significantly lower cognitive sum score, the neuropsychological results were congruent with the favourable neurological outcome found previously in several other studies on NB patients, e.g. [1–3, 35]. Our interpretation of these findings is that in the vast majority of patients, neuroborreliosis does has not have a relevant impact on their cognitive functions.

According to our data, brain atrophy as it occurs in patients after purulent meningitis [40] does not represent a common feature of neuroborreliosis. The mean brain volume and the volume of the separate brain tissues (measured automatically - thus examiner-independent) were almost identical in both groups. The high proportion of brain atrophy in patients after NB reported by Tarasow et al. [5] may be explained either by the small number of patients (*n*=14) in that study, a selection bias (the data were collected in a department of radiology which probably did not regularly receive asymptomatic patients after NB), or the fact that the patients may have been diagnosed and treated with a considerable delay after infection.

This does not mean, however, that our data rule out the possible individual occurrence of severe long-term sequelae after NB. If cognitive impairments occur, the domain of frontal executive functions (e.g. psychomotor speed, gathering and structuring of information, changing behaviour in response to changing surroundings) is the most probable to be affected after neuroborreliosis. The degree of these impairments, however, is that small that alterations in neurocognitive functioning will often be only discernible by extensive neuropsychological testing or just by the affected person him- or herself. As a consequence, assuming that our control group was representative for the study group (and we do not find any reasons against this), it can be concluded that NB may leave subtle but discernable traces for individual patients, especially concerning the frontal executive functions that only exceptionally are severe enough to affect the activities of daily living. Of course, our retrospective design bears the danger of a bias; in spite of the control group it cannot be ruled out that the pre-morbid cognitive functioning – especially the executive functions- of the NB group might have been lower than that of the control group. To falsify or confirm this hypothesis, however, would require a large population-based prospective trial with a neuropsychological evaluation of persons before and after proven NB.

Finally, it should be noted that small differences in favour of the control group might vanish with a control group consisting of former patients who had undergone hospital treatment for other non-neurological diseases, because such an event might leave its traces on a subject’s general well-being. This issue might be discussed as a possible shortcoming of the study.

## Conclusions

We found a measurable poorer cognitive functioning in the domain of frontal executive functions and for the cognitive sum score after NB while all other domains remained unaffected. The differences that we found were subtle. The patients’ brain volumes remained unaffected by NB.

This paper has been written according to the STROBE criteria.

## Abbreviations

NB: Neuroborrelliosis
SNRS: Scripps neurological rating scale
SD: Standard deviation
CSF: cerebrospinal fluid
AI: Antibody index
EM: Erythema migrans
f/m: Female/male
TAP: Testbatterie zur Aufmerksamkeitsprüfung; test battery for alertness
WMS-R: Wechsler memory scale-r
fw/bw: Forward/backward
LM: Logical memory
CVLT: California verbal learning test
VLT: Verbal learning test
ROCF: Rey-osterrieth complex figure task
LGT-3: Lern- und Gedächtnistest 3; learning and memory test 3
RWT: Regensburger wortflüssigkeitstest
FF: Figural fluency task
HAWIE-R: Hamburg-wechsler intelligenztest für Erwachsene-R; Hamburg-Wechsler intelligence test for adults-R
WCST: Wisconsin card sorting test
BDI: Beck’s depression inventory
HAMD: Hamilton depression scale
SCL-90R: Symptom checklist 90R
STAI X1/2: State-trait-anxiety inventory
FIQ: Fibromyalgia assessment questionnaire
FACIT: Functional assessment of chronic illness therapy-fatigue
hrQoL: Health-related quality of life
SF-36: Satisfaction of life scale
PSQI: Pittsburgh sleep quality index
MR: Magnetic resonance
FLASH: Fast low angle shot
